# Epidermal Growth Factor Receptor as a Model Drug Target for Scrutinizing the Status Quo in Drug Discovery and Chemical Biology

**DOI:** 10.1021/acsbiomedchemau.6c00089

**Published:** 2026-06-17

**Authors:** David E. Heppner, Monica R. MacDonald, Nader N. Nasief, Shilpa E. Naik, Sahba Cunningham

**Affiliations:** 1 Department of Chemistry, College of Arts and Sciences, The State University of New York at Buffalo, Buffalo, New York 14260, United States; 2 Department of Structural Biology, 12291 Jacobs School of Medicine and Biomedical Sciences, Buffalo, New York 14203, United States; 3 Department of Pharmaceutical Sciences, The State University of New York at Buffalo, Buffalo, New York 14214, United States; 4 Department of Pharmacology and Therapeutics, Roswell Park Comprehensive Cancer Center, Buffalo, New York 14203, United States; 5 Department of Pharmacy Practice, School of Pharmacy and Pharmaceutical Sciences, The State University of New York at Buffalo, Buffalo, New York 14214, United States; 6 National Organization for Drug Control and Research (NODCAR), Egyptian Drug Authority (EDA), Giza 12654, Egypt

**Keywords:** epidermal growth factor receptor, kinase inhibitors, structure-guided drug design, model drug target, medicinal chemistry, structural biology

## Abstract

Drug discovery scientists and chemical biologists continually seek to improve strategies for compound development. Here, we present insights derived from multiple studies of the epidermal growth factor receptor (EGFR), illustrating how this well-established target can inform practical aspects of drug discovery. Case studies spanning fragment-based drug design, bivalent inhibitor discovery, and the optimization of irreversible covalent inhibitors demonstrate how focused investigation of a single system can serve as a framework for methodological innovation and generation of broadly applicable knowledge. We propose that these collective efforts exemplify an emerging paradigm in medicinal chemistry, wherein “model drug targets” are deliberately leveraged to yield generalizable design principles and uncover new strategies for ligand discovery and chemical probe development.

## Graphical abstract

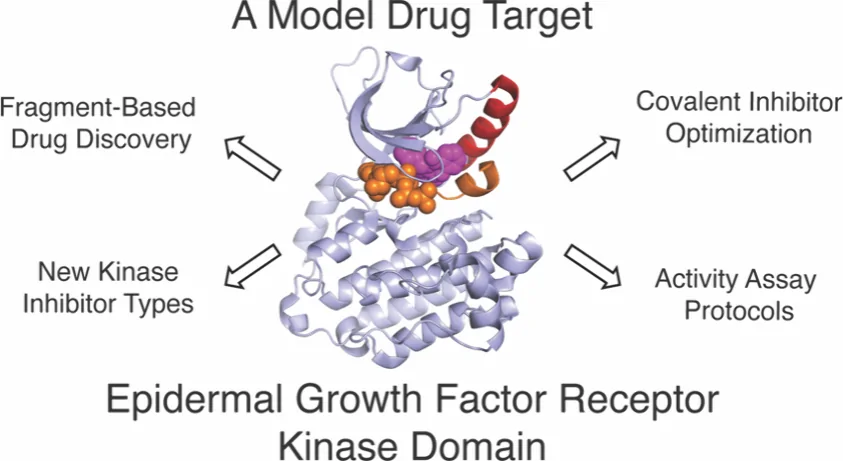

## Introduction

Researchers in chemical biology, drug discovery, and related fields all share a common goal to create molecules that establish causal links validating the involvement of protein targets in biological functions and/or disease phenotypes. The surge of interest in chemical probes have unlocked important insight into target function and, in many cases, have lent essential starting points for the discovery of novel small-molecule drugs.^1,2^ Despite the abundant successes, compound discovery and optimization remains reliant on extensive exploration. As such, improving rational design of biologically relevant compounds to enable efficient workflows in these fields is critically needed to effectively address complex problems and guide further advancement in biomedical research.

An established route in drug discovery is structure-based drug design (SBDD), a medicinal chemistry paradigm utilizing three-dimensional structural information of protein targets to iteratively optimize drug candidates.^130^ Utilizing structural models is a proven route to remove considerable ambiguity regarding protein–ligand binding resulting in an enhanced time- and cost-effective path to new therapeutics. A focus of our group has centered on the design of projects that directly address scenarios in drug discovery where guesswork and errors can potentially lead to inefficiencies in small molecule discovery. The foundation for this work has been the kinase domain of the epidermal growth factor receptor (EGFR) as an established target in oncology and the focus of extensive chemical biology, medicinal chemistry, and structural research.^3–8^

It is worthwhile stating that decades of research have laid a foundation for the rudimentary elements of drug discovery,^9–12^ including emerging subjects in knowledge sharing,^13^ refinements of “drug-likeness”,^14^ and aspects of design.^15,16^ The goal here is to deliberately address drug discovery scenarios for the development of improved workflows or frameworks that can directly influence procedures in industry and academic settings. The status quo can be scrutinized to expose avenues that complicate modern drug discovery and to offer guidance for their avoidance, while highlighting ideal approaches to rapidly design compounds with superior profile properties.

### Epidermal Growth Factor Receptor (EGFR)

The epidermal growth factor receptor (EGFR) is a master regulator of signal transduction for a wide range of cellular processes triggered by the binding of cognate ligands.^17–19^ The wide range of downstream cellular signaling cascades enabled by EGFR dimerization encompasses many phenotypes, including but not limited to cell growth, survival, and proliferation. Activating mutations within EGFR that override normal regulatory mechanisms, as well as gene amplification, exaggerate these signals and are commonly found in diverse human cancers.^20,21^ The most common tumors that harbor EGFR activating mutations include non-small cell lung cancer (NSCLC)^3,4,22^ and glioblastoma (GBM)^23,24^ where mutations are primarily located within the intracellular kinase domain or extracellular ligand binding domains, respectively.

An important attribute of NSCLC tumors driven by EGFR mutations is their dependency on EGFR signaling for proliferation and survival.^25^ The discovery of clinically efficacious tyrosine kinase inhibitors (TKIs) exposed this vulnerability and has been a mainstay in clinical oncology for over two decades.^3,4^ The first FDA-approved first-generation EGFR TKIs “Type I”, gefitinib and erlotinib, effectively target most EGFR mutant NSCLC tumors, including L858R and exon19del mutants,^26^ among others (Figure 1).^3,27^ An extended chemical scaffold is represented in lapatinib, which is a “Type I.5” kinase inhibitor due to its binding to the inactive conformation.^28–30^ In any case, these TKIs are eventually rendered ineffective by the acquisition of a highly prevalent T790M gatekeeper mutation, which strengthens the kinase domain affinity for ATP.^31^

**Figure 1:**
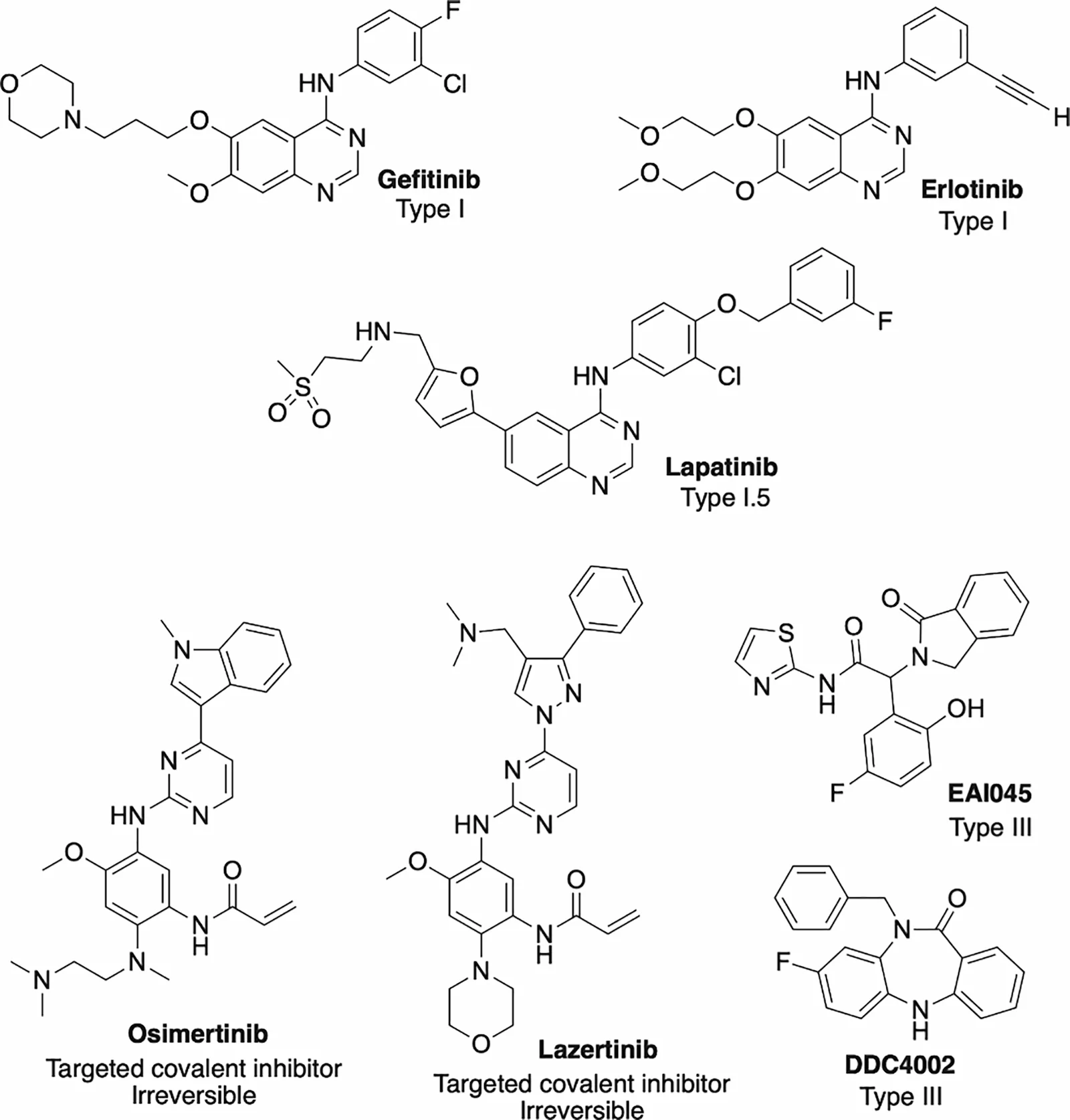
Representative chemical structures of key EGFR-targeting inhibitors.

To address T790M-positive tumors, irreversible targeted covalent inhibitors (TCI) emerged as effective treatments^32^ with osimertinib (Figure 1) initially approved as a second-line,^33–35^ but more recently shown superior for treatment-naive patients.^36^ Additionally, combination of lazertinib and dual EGFR/c-MET antibody amivantimab is also available.^37–41^ Both osimertinib and lazertinib form covalent bonds with C797 located near the ATP binding site. It has been shown, however, that these TKIs are rendered ineffective in part due to a mutation of C797 to a serine thwarting the formation of the potency-enabling covalent bonds.^42–44^

In the past decade, drug discovery has focused on developing inhibitors targeting both T790M and C797S drug-resistant mutations. Despite promising trends, no such inhibitor has gained approval.^45–58^ A unique direction concerns ATP-noncompetitive allosteric “Type III” inhibitors that bind to the inactive conformation outside the ATP binding site (Figure 1).^59–64^ Biological evidence shows that these compounds synergize with ATP-competitive inhibitors due to binding of both sites simultaneously.^61,65,66^ This has become a source of inspirations for us and others to design molecules that bind both sites.^67–131^

## Improving Understanding of Drug Discovery through Model Drug Targets (MDT)

While all drug discovery campaigns are unique, they all share the common goal of identifying leading compounds on efficient timescales. The multiparameter nature of drug discovery naturally manifests ambiguous pitfalls that can result in wasteful pursuits.^74^ One route to efficiency is to eliminate choices with constraints to chemical compositions. Medicinal chemistry heuristics provide approaches to these guidelines such as the “Rule of Five”^75^ and others for maintaining “druglikeness.” ^14,76^ Notably, another editorial states “··· The best advice for a medicinal chemist is to do your absolute best to design studies/assays to kill your compound, to kill the series, and to kill the target/program. If you are not able to do so, then you have a compound and a program that may move smoothly through development.”^77^ Indeed, self-scrutiny can be one of the best defenses to mitigate serious problems often encountered upon further investigation.

Another route to accelerating drug discovery is to scrutinize scenarios toward improved understandings and/or expose weaknesses in an approach. As such, proteins can be recognized as Model Drug Targets (MDTs) to offer guiding principles in studies that would serve the community through experiments aiming to refine knowledge in certain drug discovery or chemical biology scenarios. Importantly, an MDT study of a scenario may also be pursued in the context of a modern drug discovery campaign allowing for the generation of broader concepts. It is important to state that any one MDT is not expected to apply to all drug discovery scenarios, and considering studies from diverse MDTs is likely important for generalizing procedures or workflows.

With our focus on EGFR, several attributes and properties have become apparent in enabling the identification of EGFR as an MDT (Table 1, Figure 2). One key attribute is for there to exist a diverse repertoire of small molecules targeting the MDT. The sizable arsenal of EGFR TKIs consists of ATP-substrate competitive and noncompetitive allosteric pocket inhibitors, in addition to covalent binding agents.^6^ Another essential property for the MDT is to be structurally enabled through atomic-resolution experimental methods where scenarios in drug discovery related to compound design are best addressed.^68,71,73^

**Figure 2:**
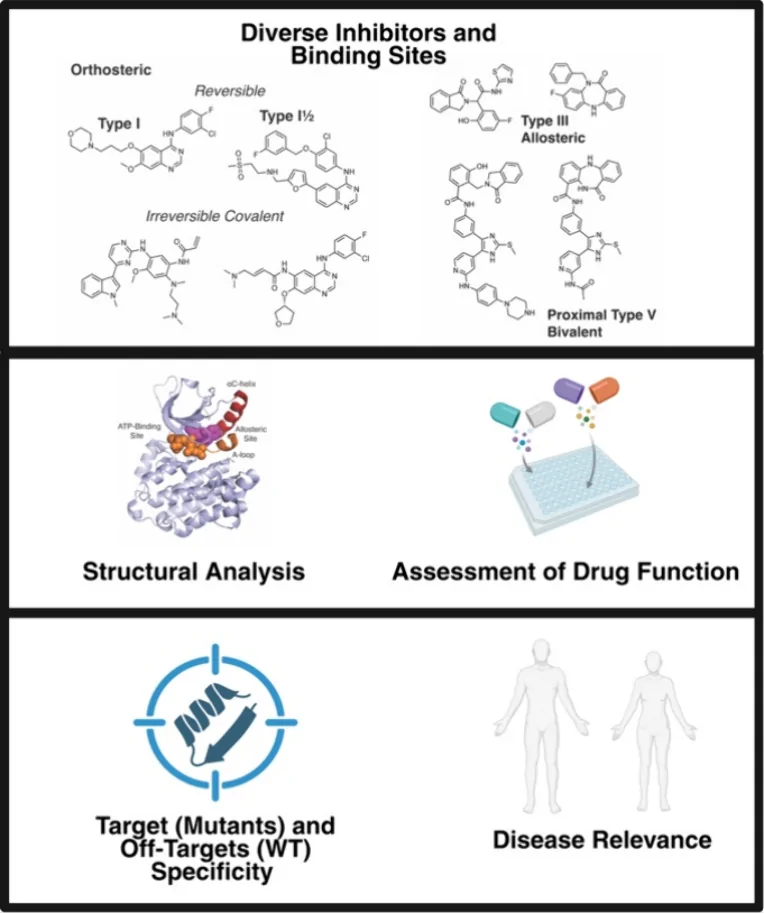
Attributes of a model drug target (MDT). Characteristics that make the EGFR kinase or another protein an MDT include diverse inhibitors that can bind to the active site, the ability to gain high-resolution structures for ligand binding analysis, and the ability to assay biochemical and cellular properties. This provides the capability to assess target specificity as well as disease relevance.

**Table 1:** Attributes of an MDT and the Impact of EGFR Studies on Drug Discovery

Attributes of an MDT	Demonstrated by EGFR	Drug Discovery Scenario	Specific Framework or workflow
Diverse Tools: Biochemical Activity Assays Available	Recombinant constructs (commercially available). Highly sensitive tyrosine kinase continuous and end point assays available for inhibitor characterization at low picomolar enzyme concentrations.	Activity Assay Protocols, Structure-kinetic Relationships, Structure–activity Relationships, and comparison to literature	Covalent Inhibitor Methods,^100^ Optimization Workflow.^78^ Target (e.g., EGFR Mutant) Selectivity.^68,70,78^
Diverse Assessments of Drug Function: Cell line models available	Various EGFR expressing lines and engineered Ba/F3 cells available for activating mutations and WT where proliferation is coupled to EGFR targeting.	Correlation to biochemical activity assays	Covalent Inhibitor Methods,^100^ Optimization Workflow.^78^ Target Validation with no established biochemical assay.^70^
Disease Relevance and Approved Drugs	Oncogenic driver of NSCLC and other tumors	Correlation of biological findings to clinical needs.	Present new preclinical candidates.^70,71^
Structurally Enabled (Pocket architecture and regulation)	X-ray cocrystal structures	Structure-guided design	New linker optimization design in fragment-based drug discovery,^71^ Structural basis for selectivity.^70^
On/off targets	Activating mutations as targets and WT as an off target.	Factors influencing compound Selectivity	Covalent Inhibitor Optimization,^78^ Expanding kinase inhibitor types

Methods to assess compound potency in both cellular and biochemical assays are absolutely required. Research with both settings highlight scenarios where cellular effects do not correlate with biochemical potencies and thus, potentially misguide a project. A somewhat unique aspect of EGFR as an MDT is the requirement for mutant-selective potency to establish an effective therapeutic window for NSCLC drug candidates. This creates a set of “targets” (EGFR mutants) and an “off-target” (WT) that is useful, especially as seen for targeted covalent inhibitor optimization^78^ and identifying pockets for mutant-selective binding.^70,73^ Finally, an MDT that is relevant in diseases enables the development of potential translational agents.

An MDT study is worth comparing with earlier works that provided foundational knowledge of protein–ligand binding.^9^ One such example concerns thermolysin,^79^ that led to foundational knowledge of isosteric replacements,^80^ roles of hydrophobic effects, and solvent waters.^81–87^ Another protease, thrombin, served as a useful platform to explore nonadditive (cooperative) binding,^88–90^ ligand desolvation^91,92^ and protonation state.^93^ Carbonic anhydrase^94^ has additionally served as a models for understanding hydrophobic effects,^95,96^ water restructuring,^97^ and inhibitor thermodynamics.^98,99^ Details at this level inspire comparable avenues for other drug discovery projects, but do not necessarily improve decision making within practical contexts. In this way, an MDT study can be considered unique from these pioneering studies.

It is also useful to consider how to frame an MDT study (Figure 3). The emphasis should be placed on a certain drug discovery scenario. A clear example of such would be an aspect of structure optimization,^68,70,71,73^ assay methodologies,^100^ and/or the generation of frameworks for interpreting functional data.^78^ Hypotheses should be framed so that experiments can confirm or refute the hypotheses and be further supported by subsequent studies until a clear framework (i.e., a basic structure underlying a concept) or efficient workflow (i.e., sequence of events or other processes through which a piece of work passes from initiation to completion) is meaningfully achieved. Special attention should be paid to instances where predictability is challenging, which are potentially the cases for rejected hypotheses, and should inform the framework or workflow, providing context to the next steps in optimizing drug potencies and interactions. Another important instance of a rejected hypothesis may arise when the accumulated data present unexpected results that may be due to an serendipitous variables. As such, accumulated insights toward generating frameworks or workflows will provide guidance for drug discovery scientists, and thorough studies across various MDTs ultimately generate increasingly robust processes.

**Figure 3:**
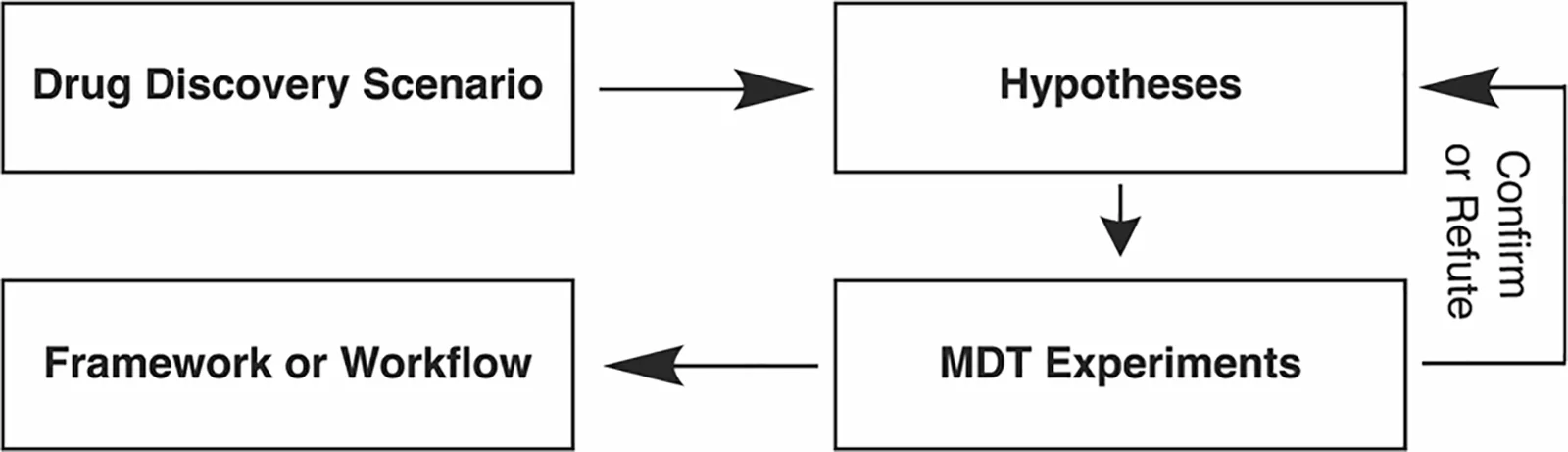
A generalized approach to using an MDT in the context of generating a framework or workflow in drug discovery.

### Binding the ATP and Allosteric Sites: Rational Design with Unexpected Insights

Initially motivated by studies showing cooperative effects and cobinding of ATP-competitive and allosteric EGFR inhibitors,^61,63,65,66^ we sought to design molecules that simultaneously occupy both pockets. Recently reported structural findings of a series of trisubstituted imidazoles served as ATP-site starting points (Figure 4A)^45–48^ where overlapping features between the LN2084 and EAI045 enabled iterative structure-guided design (Figure 4B–D).^48,59,60^ Due to their dual pocket binding modes, we denote these molecules as “ATP-allosteric bivalent inhibitors” (AABIs).

**Figure 4:**
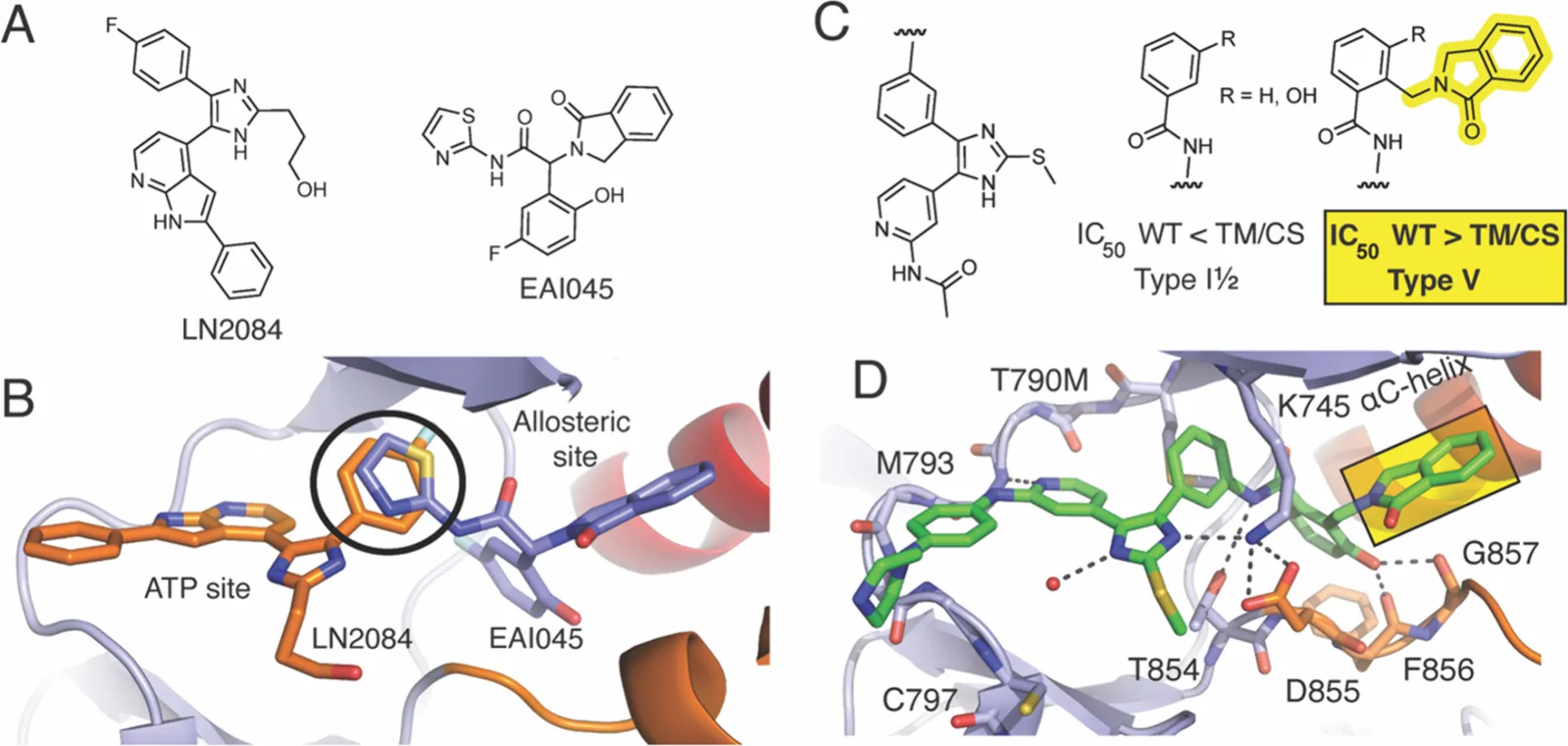
The rational and structure-guided design of ATP-allosteric bivalent inhibitors (AABIs) targeting EGFR. (A) Chemical structures of ATP-site binding imidazole (LN2084) and allosteric inhibitor EAI045. (B) Overlay of X-ray cocrystal structures of LN2084 (left, PDB ID 6 V5H) with EAI045 (right, PDB ID 6P1L) with overlapping rings highlighted with a circle. C) Summary of the SAR of EAI045-inspired AABIs where a methyl isoindolinone is responsible for enabling a mutant-selective “proximal Type V” profile. D) X-ray cocrystal structure of LN5461 as an example of a reversible binding “proximal Type V” AABI with the selectivity-enabling group highlighted in the transparent yellow box (PDB ID 8TO3). Reproduced or adapted with permission from ref 70. Copyright 2024 American Chemical Society.

Initial proof-of-concept molecules consisted of a 1,3-dioxoisoindolin-2-yl (phthalimide) stemming from a fluorophenol with a “linear” branch.^68^ Desiring to better understand the potency and mutant selectivity of these inhibitors, we underwent an SAR campaign where it was discovered that certain allosteric pocket groups either strongly targeted WT or drug-resistant mutations (Figure 4C).^70^ Specifically, the binding of a substituted phenyl ring within the “back pocket” improved potency but was not mutant selective.^68,70,73^ However, upon further incorporation of a methylisoindolinone mutant EGFR becomes selectively inhibited compared and WT to a lesser extent (Figure 4C). The most optimal AABIs, including reversible and covalent analogues, exhibit this mutant-selective profile in association with the presence of the extended groups that bind within the allosteric site (Figure 4D), which establishes these AABIs as unique compared to others reported in the literature.^67,69,72^

We next desired to determine the most appropriate “type” for these AABIs.^6,101^ Initially, it seemed that the most likely option would be “Type 1.5” based on their expanded structrures similar to lapatinib.^70^ However, the imidazole-based inhibitors extending into the “back pocket” fail to inhibit T790M, which is not the case for our AABIs.^70,73^ Cellular studies further distinguish our AABIs from lapatinib by their activity against E746-A750 exon19del, which are insensitive to inhibition by both “Type 1.5" and “Type III” allosteric inhibitors.^70^ Therefore, the designation of “Type 1.5” and “Type III” is inappropriate. Since the ATP and allosteric sites can be considered separate binding pockets, as already discussed, we noted their binding as “bivalent” in nature, which is consistent with “Type V”. Rare examples of bivalent kinase inhibitors are knownand most commonly explored in the context of compounds that bind sites at significant distance (>20 Å), whereas these AABIs bind to adjacent pockets.^102^ We describe the mutant-selective AABIs as “proximal Type V” to indicate their unique profile as well as the spacial location of the targeted sites.^70^

### Refocus toward Fragment-Based Drug Design

While pursuing a thematically similar project, an unexpected application for EGFR AABIs originated from incorporating a dibenzodiazepinone (benzo) moiety based on DDC4002 (Figure 5A).^60^ The resulting *N*-linked molecule did not exhibit meaningful biochemical potency and an X-ray cocrystal structure provided some guidance as to why (Figure 5B).^71^ A redesign was aimed to “revert” the conformation of the benzo and was accomplished through the production of a *C*-linked analogue (Figure 5A,C).^68,70,73^ Surprisingly, this compound was exceptionally more potent representing a ∼ 1-million-fold improvement in EGFR kinase inhibition.^71^ The binding features at the ATP site were identical while distinct conformations were observed in the allosteric pocket with the *C*-linked compound best matching the parent DDC4002 allosteric inhibitor (Figure 5D).^71^

**Figure 5:**
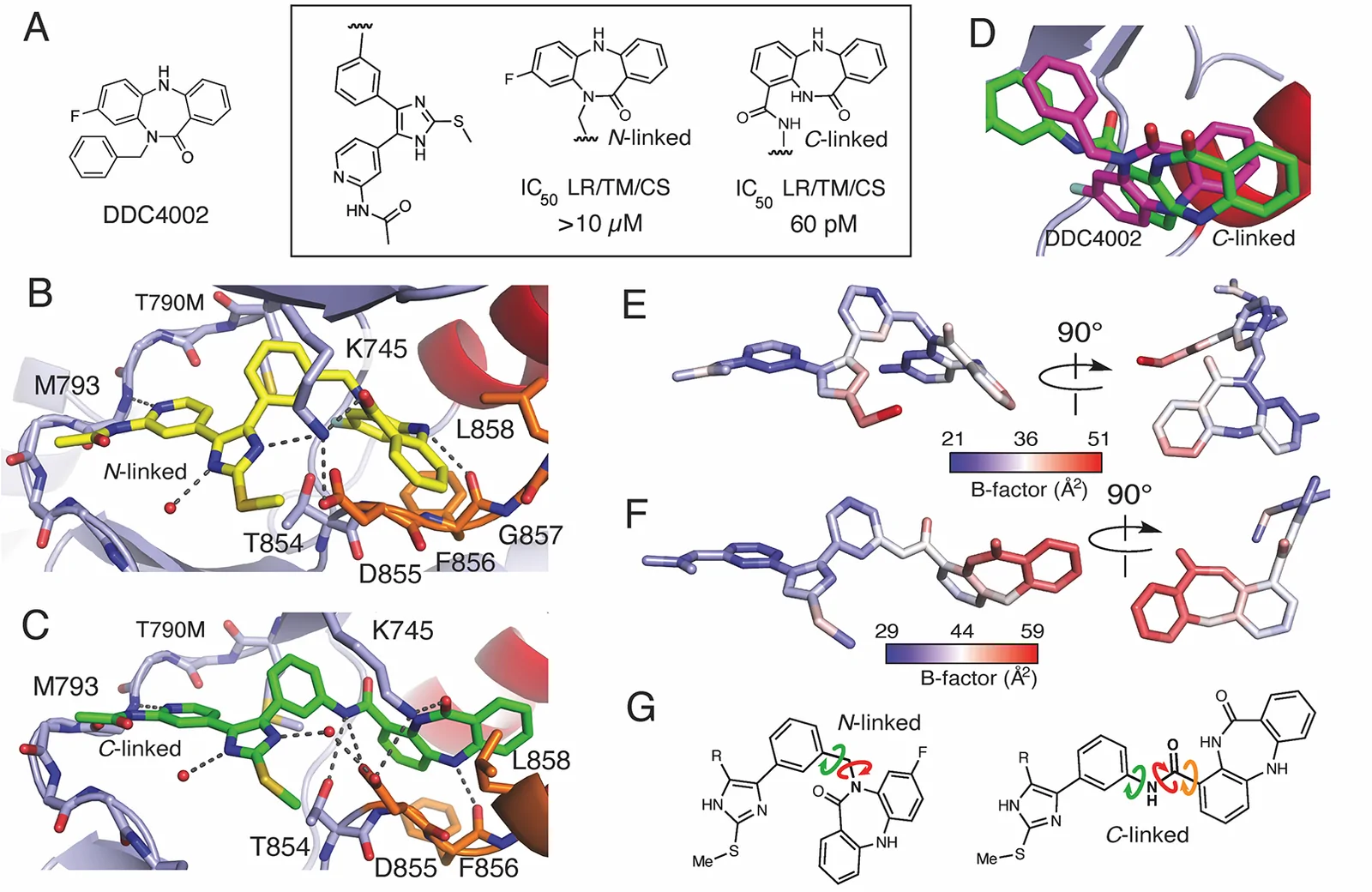
Structural changes to the linker region of benzodiazepinone (benzo)-derived AABIs results in binding mode changes consistent with large differences in potency. (A) Chemical structures of benzo allosteric inhibitor DDC4002, along with two benzo-containing AABIs (in box), one of which exhibit >10^6^-fold difference in potency for L858R/T790M/C797S (LR/TM/CS) mutation (*C*-linked). (B) Binding mode of the *N*-linked compound showing “outward” benzo butterfly (PDB ID 8FV3). (C) Binding mode of the *C*-linked compound with “inward” benzo conformation (green, PDB ID 8FV4). (D) Overlay of cocrystal structures showing the *C*-linked compound (green, PDB ID 8FV4) more closely matching the binding mode of DDC4002 (magenta PDB ID 6P1D). Representation of atomic B-factors for (E) *N*-linked and (F) *C*-linked compounds. (G) Linker torsion angles for both molecules highlighting the additional C–C torsion angle enabling enhanced flexibility for the *C*-linked compound.

Reprinted or adapted with permission under a Creative Commons 4.0 from ref 71. Copyright 2024 Nature Portfolio.

Since these two molecules are strikingly different in potency, and only structurally distinct with respect to the linking groups, we sought to answer: “What molecular factors distinguish the linkers in terms of their ability to make an AABI maximally potent?” This question is broadly relevant to fragment-based drug discovery (FBDD) as linking fragments together is often unsuccessful.^103,104^ Searching for potential explanations, one notable observation contained within these X-ray cocrystal structures was the trend in the crystallographic B-factors, which reflects the degree of atomic mobility and positional uncertainty within a structure.^105^ The atomic B-factors indicated that the *C-*linked compound features more flexibility compared to the *N*-linked molecule stemming from the amide linker (Figure 5E,F).^71,104^ Molecular dynamics (MD) simulations provided additional information, as they predicted that the two compounds bind with a large differences in affinity with the *C*-linked compound binding with greater H-bonding and van der Waals interactions.^71^ Other key properties from calculations indicated that the *N*-linked compound adopts an energetically unfavorable conformation while the *C*-linked compound is bound in an energetically favorable conformation. The decisive piece of evidence came from analysis of the torsion angles of the rotatable bonds of the two linkers (Figure 5G). The key functional attribute that results in superior binding of the *C*-linked compound arises from one unique rotatable C–C bond of the benzo phenyl ring (orange in Figure 5G). This rotatable bond allows for *enhanced* flexibility enabling the allosteric benzo to adopt the most favorable binding mode. It seems clear that these insights were only possible through utilizing experimental structural techniques, where correct physical poses and alternative side chain positions were observed.^71^ We speculate and further reinforce the need for experimental data in MDT studies, as many of these details would likely have been missed in an AI-generated or *in silico* structure-ligand models.^106^

The work above on AABIs offers unique insights, where generalizable applications to drug discovery are derived through utilizing EGFR as an MDT (Figure 6). The striking preference for mutant selective inhibition of our “proximal Type V” AABIs pinpoints the allosteric pocket as the key binding site responsible for this highly sought after selectivity profile.^70^ This observation, compared to “Type II” or “DFG-out” inhibitors, shows distinct binding of the AABI within the allosteric pocket, specifically an open channel made accessible through rotation of the αC-helix “out” in the inactive conformation (Figure 6A). Important drug properties often associated with “Type II” compounds, which bind in a distinct pocket made accessible by rearranging the DFG-motif (i.e., the “DFG-out-channel”), suggest similar effects can be achieved in the context of “proximal Type V” (Figure 6A). Our structural searches for kinase inhibitors that bind within an “αC-helix-out channel” are significantly underexplored and identify this site as a new direction in kinase inhibitor development.^70^ This example highlights how EGFR can serve as an MDT that reveals insights into new drug target pockets to enhance binding properties and provide a platform for expanding the arsenal of inhibitors in a drug target class.

**Figure 6:**
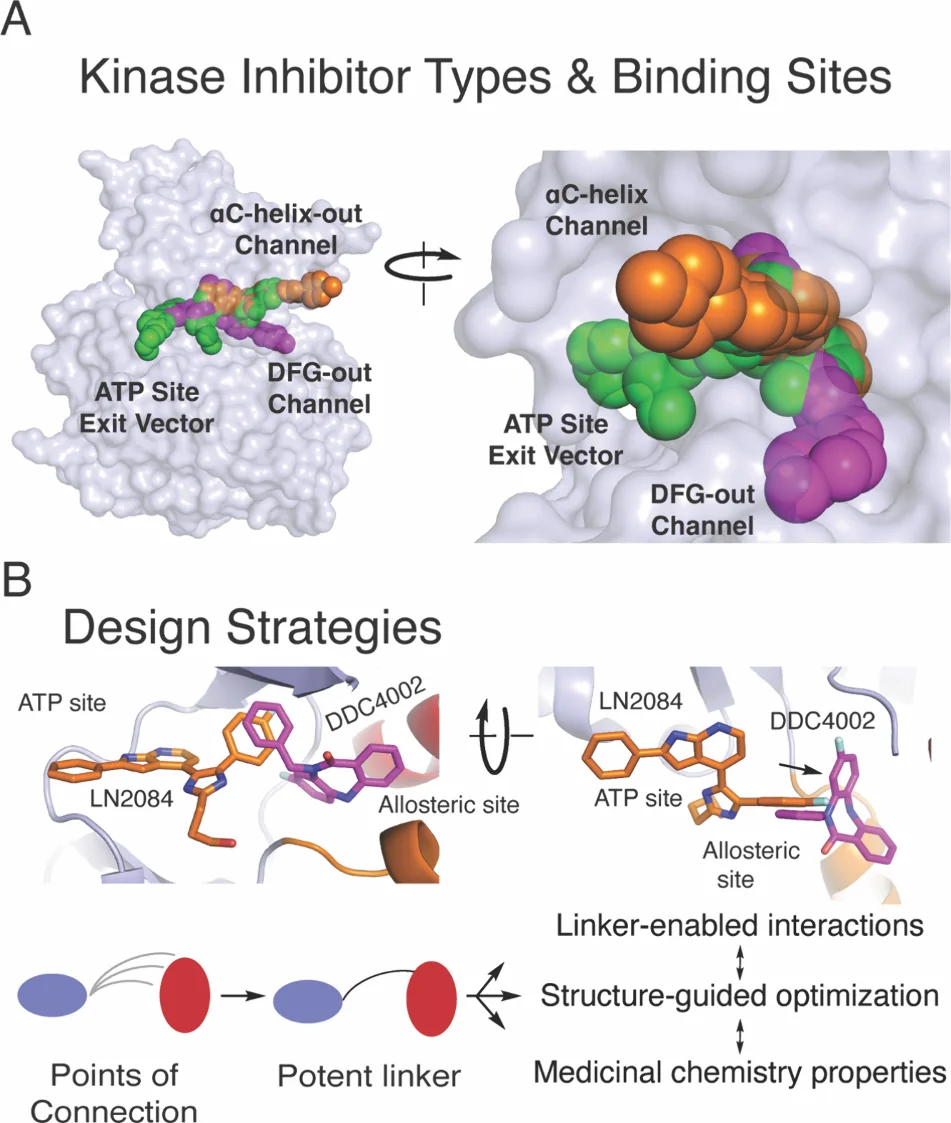
Discovery of EGFR AABIs enables distinct insights into drug discovery. A) “Proximal Type V” inhibitors highlight the importance of binding pockets of the inactive conformation underexplored in kinase inhibitors. Orange desribes the binding of limitedly explored "Proximal Type V" inhibitors while magenta showcases the binding of more common "Type II" inhibitors and their respective channels of the inactive kinase domain. Reproduced or adapted with permission from ref 70. Copyright 2024 American Chemical Society. (B) Benzo-derived AABIs produce new design strategies in bivalent inhibitor optimization and promote highly effective “points of connection” optimization to atoms of certain fragments that are not readily apparent from simple structural overlays. For instance, the “overlapping” phenyl rings between LN2084 (PDB ID 6V5N) and DDC4002 (6P1D) would likely inspire explorations of linkers along this *N-*linked vector and not the *C*-linked position (arrow in top-down representation).

Reprinted or adapted with permission under a Creative Commons 4.0 from ref 71. Copyright 2024 Nature Portfolio.

The example of the two disparagingly potent AABIs discussed previously enabled a new design strategy in FBDD (Figure 6B).^71^ Given the relatively limited success in fragment linking,^103^ insights into the mechanisms of the *N*- and *C-*linked inhibitors pointed toward first optimizing “points of connection” to ensure maximal improvements in binding.^71^ Given this, it is informative to pose the question, “When considering the binding modes of the original inhibitors, would the *C*-linked compound have been an obvious starting point?” A top-down view of the binding shows the positioning of the *C*-linked carbon versus the overlapping phenyl rings of LN2084 and DDC4002 would inspire extensive exploration of *N*-linked scaffold (Figure 6B). Admittedly, had we strictly followed structural overlay, we would have missed the opportunity of discovering the *C*-linked molecules. By linking the two groups at different points of connection, the linker can alter the flexibility of the two groups allowing for dramatic enhancements in potency. After finding the most favorable point of connection, we rationalize that more classical optimization procedures would follow. This example corroborates how EGFR as an MDT can lead to streamlined approaches and novel design strategies in drug discovery.

### Targeted Covalent Inhibitors (TCIs)

The development of enzyme inhibitors that form irreversible covalent bonds with their targets is increasingly commonplace in chemical biology and medicinal chemistry.^107^ Initially championed by B.R. Baker,^108^ rationally designed covalent inhibitors, reached a watershed moment with the FDA approval of afatinib targeting EGFR and ibrutinib targeting BTK in 2013 for oncology indications.^107,109–111^ Importantly, a compound with otherwise weak reversible binding can be made into a highly efficacious agent, even in the context of relatively slow covalent bond formation.^112^ This has expanded the scope of drug discovery in several ways, including enabling the targeting of drug-binding sites that are relatively shallow and increasingly utilized to target unstructured regions in conventionally undruggable proteins, such as transcription factors or targets involved in protein degradation.^113–115^

An important distinction concerning irreversible TCIs from the conventional reversible binding inhibitors is the requirement for their potency to be determined in time-dependent activity assays.^100,116^ This is because TCIs operate through a two-step mechanism (Figure 7) where inhibitors first bind the enzyme in a fully noncovalent complex (E·I) and then irreversibly form the covalent bond to form the permanently inactivated enzyme (E–I). The kinetic parameters utilized in the characterization of these agents are the inactivation efficiency (*k*_inact_/*K*_I_, described as the second-order rate of enzyme inactivation) and the inactivation constant (*K*_I,_ described as the concentration of inhibitor that yields an observed rate constant of inactivation of 1/2 *k*_inact_). The comparison of second-order *k*_inact_/*K*_I_ rates is the most accurate and reliable measurement of relative potency among molecules, which can be utilized to assemble “structure-kinetic relationships” (SKRs), analogous to structure–activity relationships (SARs) conventionally reported for reversible-binding (non-covalent) compounds. It is helpful to acknowledge that persistent issues exist in the literature regarding covalent binders, especially with respect to the erroneous reporting of kinetic terms.^100,116^

**Figure 7:**
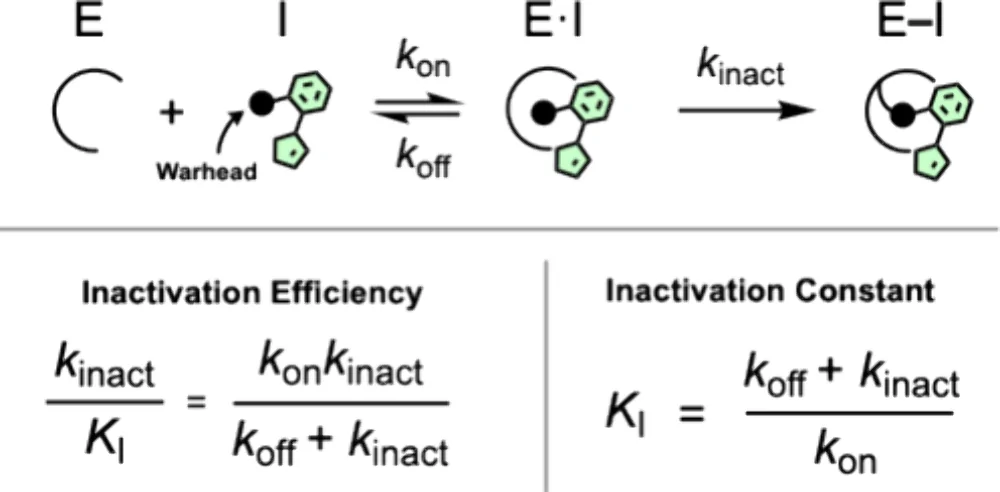
Two-step mechanism of irreversible inhibition of an enzyme by a covalent inhibitor and related kinetic terms utilized in evaluating their potency and selectivity. Reproduced or adapted with permission from ref 78. Copyright 2025 American Chemical Society.

Our earliest studies on covalent inhibitors stemmed from characterizing YH25448 (lazertinib).^38,40^ Initial *k*_inact_/*K*_I_ values assisted in the interpretation of interactions seen in X-ray cocrystal structures; however, follow up time-dependent activity assays generated significantly different rates caused by an alteration in inhibitor solution preparation.^100^ Specifically, TCIs initially prepared in 10% DMSO stocks showed consistently slower *k*_inact_/*K*_I_ values compared to those prepared from 100% DMSO stocks, despite the consistent dilution to a final 1% DMSO in both cases (*k*_inact_/*K*_I_ rate changes are consistent with earlier work).^100^ However, the variations in kinetic values appeared to be unique to the specific inhibitor. This is troublesome and highlights an unexpected pitfall where *potency measurements change unpredictably depending on how the covalent inhibitors are initially handled* *despite being assayed with identical final conditions*. In this case, the measured *k*_inact_/*K*_I_ rates could not be compared unless protocols are strictly consistent. While more research is needed to fully appreciate the origin of these effects, this observation highlights how EGFR can function as an MDT, revealing potential pitfalls in potency assays that impact decision making in drug discovery. This is one of several properties of covalent inhibitors that we have analyzed and provided guidance concerning a variety of assay conditions and comparisons to the literature.^100^

Over the course of our studies of lazertinib and osimertinib,^37,38,40,100^ we became increasingly interested in the interpretation of *k*_inact_/*K*_I_ values. To this end, we employed EGFR as an MDT for assembling rules including a discovery workflow derived from the data of 14 EGFR TCIs in biochemical activity assays (*k*_inact_/*K*_I_) and antiproliferative potency (EC_50_) values across a variety of mutant EGFR cell line models (Figure 8A).^78^ We discovered that faster L858R/T790M (LR/TM) biochemical *k*_inact_/*K*_I_ values were largely consistent with lower (more potent) EC_50_ values in H1975 cells across orders of magnitude in both cases. This was also the case for parallel biochemical *k*_inact_/*K*_I_ values against WT EGFR in relation to EC_50_ values against A431 cells; however, the effects of the studied set of TCIs showed opposite preference for WT compared to the LR/TM mutation (Figure 8B). This observation proved that *k*_inact_/*K*_I_ values increase in orders of magnitude, consistent with significant improvements in cellular antiproliferative potency.

**Figure 8:**
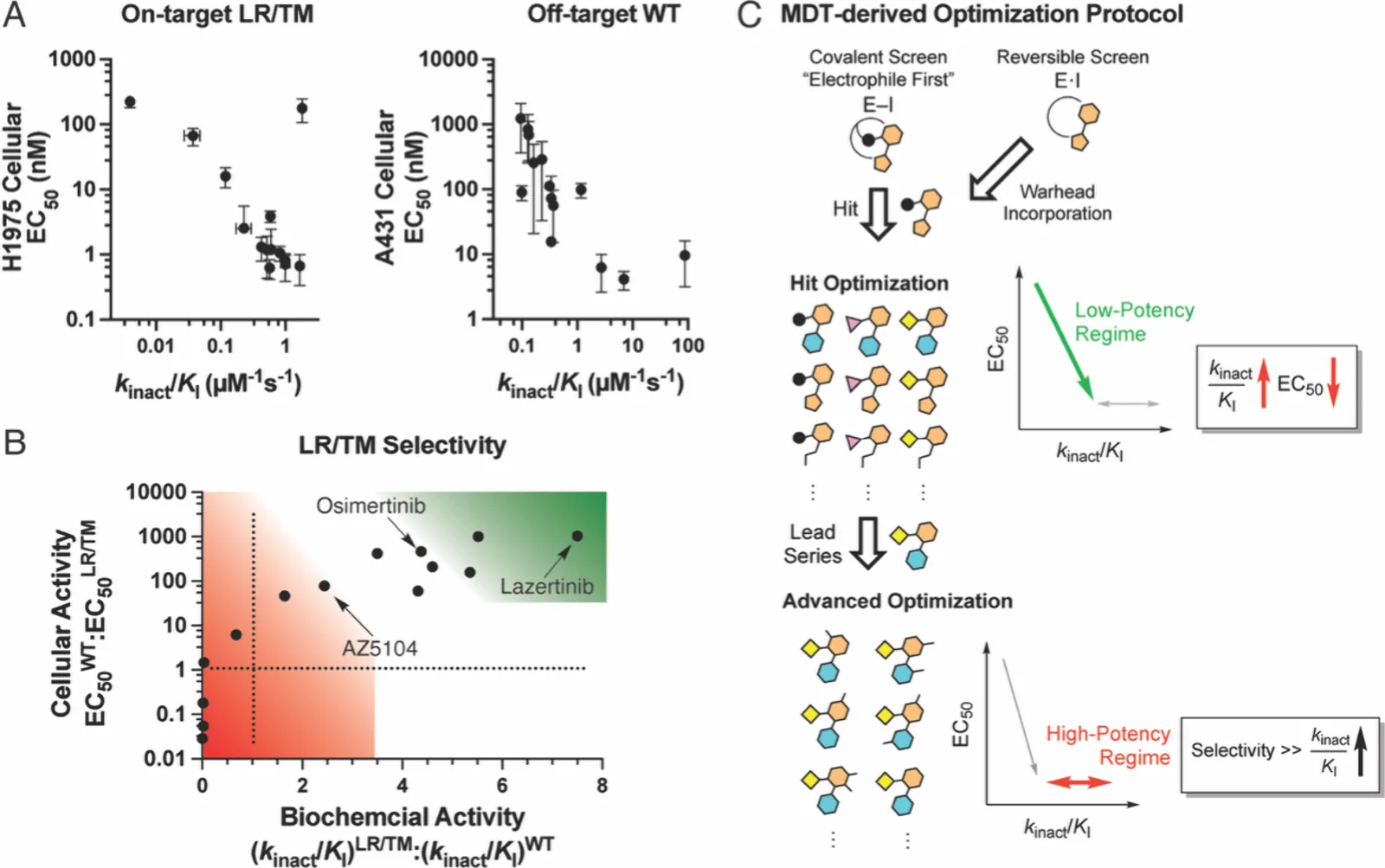
(A) Correlation of cellular antiproliferative activity and biochemical enzyme inactivation efficiency rates for “on-target” LR/TM and “off-target” WT EGFR. (B) Plotting the LR/TM selectivity in both cellular and biochemical settings, effectively rank promising TCIs. (C) Resulting workflow from EGFR MDT experiments, showcasing how enzyme inactivation efficiency values can be used in compound optimization. See original report for full details on compounds utilized in these plots. Reproduced or adapted with permission from ref 78. Copyright 2025 American Chemical Society.

Further underscoring the power of using EGFR as an MDT relates to the scenario of “decision making” in TCI discovery. If presented with the activity data in Figure 8A, it is not simple to determine which compound(s) exhibit the best properties among the series of 9 most potent TCIs. One would expect the fastest TCI to be the best; however, we intentionally complicated this analysis by including AZ5104, an active metabolite of osimertinib that is associated with undesired effects.^37,117^ We interpreted this challenge with the need to expand the profile of TCIs beyond on-target LR/TM profiling and thus incorporated a plot to rank order compounds on the basis of their mutant selectivity (Figure 8B). This representation led to a clearer ranking of TCIs, highlighting that increasingly faster *k*_inact_/*K*_I_ values may not necessarily indicate the best TCI (Figure 8B).^78^

These accumulated observations enabled an improved workflow for the development of TCIs (Figure 8C).^78^ It is clear that TCI optimization should be initially carried out to prioritize molecules with increased *k*_inact_/*K*_I_, expecting cellular potency to improve due to enhancing weak noncovalent interactions. This procedure is well aligned with the increasingly prevalent “electrophile first” discovery protocols, which emphasize the search for small fragments incorporating diverse electrophiles that have weak reversible binding affinities.^118–120^ These findings show that increasingly faster *k*_inact_/*K*_I_ values may not lead to apparent improvements in cellular activity, and our results recommend that an expanded profile is best for rank ordering TCIs in lead optimization. Importantly, we noted that LR/TM selectivity was afforded through modest fold-changes in *k*_inact_/*K*_I_ indicating that critical functional properties can be enabled through subtle changes in TCI kinetics.

## Conclusions and Perspective

The concepts raised in this perspective, where drug discovery can be improved through modeling of certain scenarios by MDT studies, offer a unique platform to better appreciate where improvements should be made. Our work on EGFR has impacted scenarios that are either mainstays of EGFR drug development and others that have been limitedly explored against this target. In several respects, the MDT needs to serve the tested scenario first, which may not necessarily fall within the established target context.

The work detailed here represents only a small fraction of the diverse scenarios often encountered in drug discovery. While we have found useful insights into compound design, assay techniques and others, many aspects of medicinal chemistry remain underexplored. MDTs that can speak to drug-like properties, such as oral availability, blood-brain barrier penetrance, and other pharmacokinetic properties, are critically needed. In addition, many questions persist in emerging areas such as addressing unstructured protein targets,^121^ induced proximity,^122^ and targeted protein degradation.^123,124^ What seems of unique timeliness is the understanding of protein–ligand docking and AI deep-learning, with the growing emphasis on deploying these computational methods toward predicting drug properties.^125^ Growth of MDT studies should be positioned toward scrutiny of these emerging techniques to gain a full understanding of their capabilities and how to best deploy them universally.

Another area of growth required is to expand the arsenal of protein targets as MDTs. While EGFR has been highly informative, it is inherently limited. One such aspect regards the nature of the drug-binding pocket, which is occluded within a catalytic cleft. Many drugs have been developed in these deep structured pockets, and more attention should be paid to highly sought-after but challenging shallow or weak-binding sites.^112,126^ To search for these opportunities, exploring new MDTs is a crucial next step, and may require the desertion or acquisition of novel attributes compared to those mentioned above (Figure 2, Table 1). For example, we have recently utilized a NADPH Oxidase (NOX) 5 protein system as an MDT to unlock previously underappreciated ways to produce selective and potent covalent inhibitors based on nucleophilic aromatic substitution warhead mechanisms.^127^ It is likely that all drug discovery projects focused on well-characterized targets can serve as MDTs. The field should direct attention toward conventionally “difficult to optimize” and “undruggable” scenarios to accelerate the development of needed therapies. A collective effort to diversify and expand MDT studies is likely to have lasting impacts on drug discovery and chemical biology.

## Abbreviations

AABIs: ATP-allosteric bivalent inhibitors
benzo: Dibenzodiazepinone
DMSO: Dimethyl sulfoxide
EGFR: Epidermal growth factor receptor
exon19del: Exon 19 deletion
FBDD: Fragment-based drug discovery
GBM: Glioblastoma
*K*_I_: Inactivation constant
*k*_inact_/*K*_I_: Inactivation efficiency
LR/TM: L858R/T790M
MDTs: Model drug targets
MD: Molecular dynamics
NOX: NADPH Oxidase
NSCLC: Nonsmall cell lung cancer
SARs: Structure–activity relationships
SKRs: Structure-kinetic relationships
SBDD: Structure-based drug design
TCIs: Targeted Covalent Inhibitors
TKIs: Tyrosine kinase inhibitors
WT: Wild-type
